# Supplementary material for: Editing of the TRIM5 Gene Decreases the Permissiveness of Human T Lymphocytic Cells to HIV-1
Source: Viruses. 2020 Dec 25;13(1):24. doi: 10.3390/v13010024 (PMC7824555; doi:10.3390/v13010024)
Supplement: Supplementary file 1 [file viruses-13-00024-s001.pdf]

Désaulniers et al.

Editing of the TRIM5 gene decreases the permissiveness of human T lymphocytic cells to HIV-1

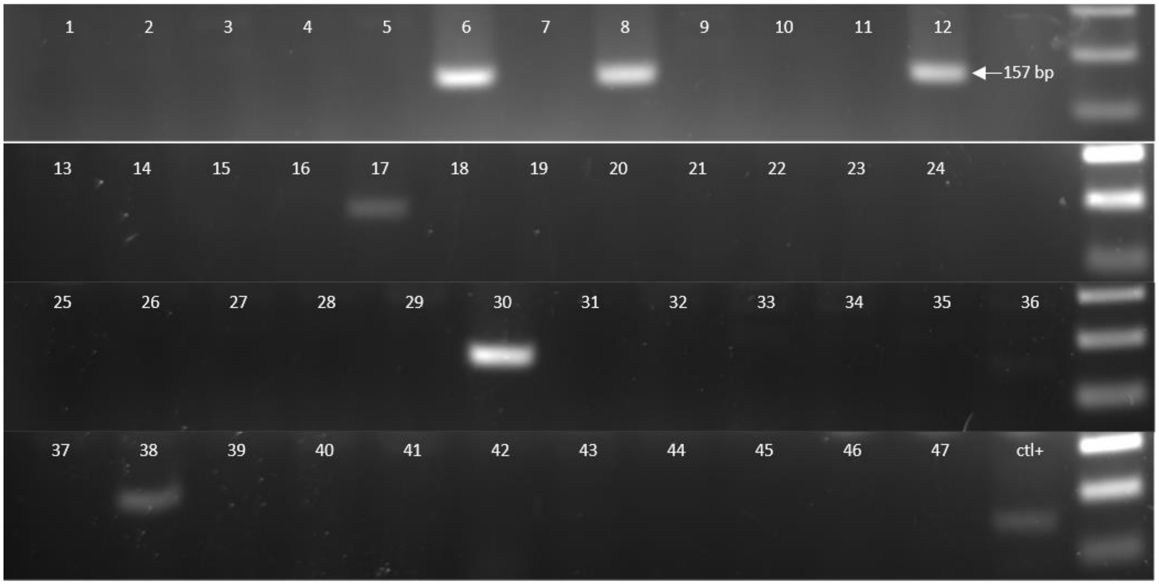

Figure S1. HDR editing-specific PCR screening of Jurkat cells transfected with CRISPR-Cas9 RNPs and donor ssDNA. 47 clonal cell populations were lysed and subjected to a PCR assay in which one of the primers is complementary to the correctly HDR-edited *TRIM5* region targeted for mutagenesis. A PCR product of the expected size was found in clones 6, 8, 12, 17, 30, 38. Ctl+ consisted of Jurkat cells following CRISPR components transfection but prior to the isolation of clones.

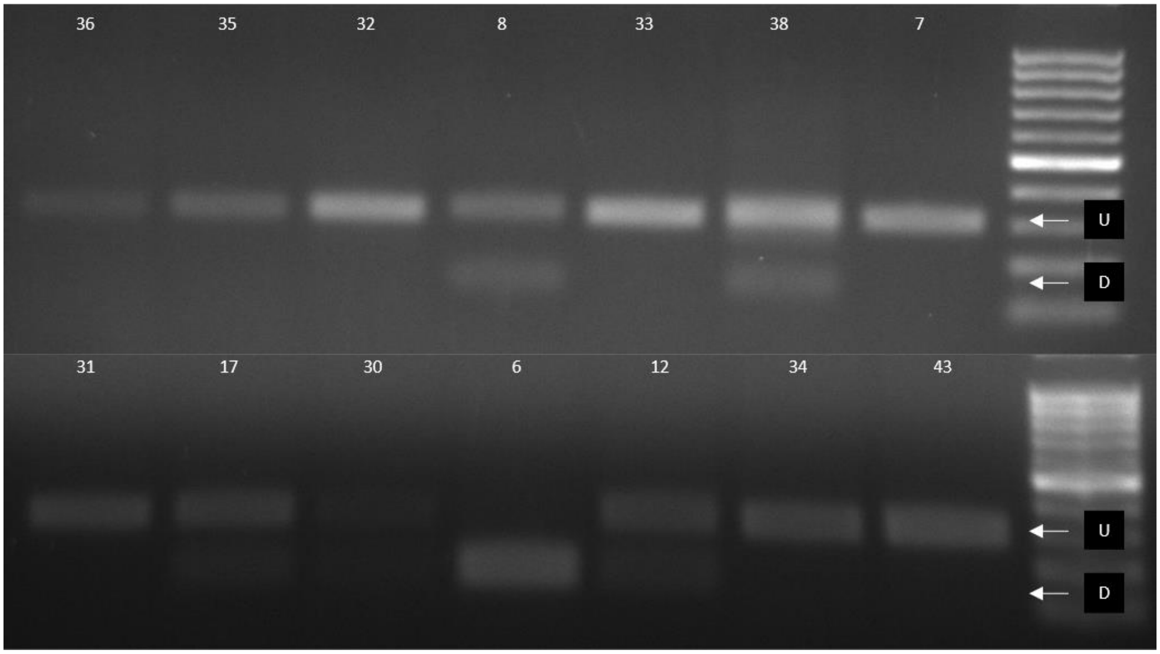

Figure S2. HaeIII screening of selected Jurkat clones. The 6 clones found to be positive in the HDR editing-specific PCR test, along with 8 randomly chosen negative clones, were subjected to a PCR assay using primers that bind outside of the genomic region complementary to the HDR donor DNA, followed by digestion with HaeIII. U and D indicates bands of the expected size for the undigested PCR product and the HaeIII digestion products, respectively.

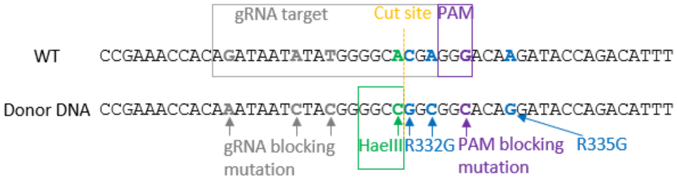

		N mutations	Indels	Subst.
**Clone6**	CCGAAACCACAGATAATATACGGGGCCGGCGGCACAAGATACCAGACATTT	5/8	n	n
	CCGAAACCACAGATAATATATGGGGCCGGCGGCACAGGATACCAGACATTT	5/8	n	n
Clone 7	CCGAAACCACAGATAATATATGGGGCACGAGGGACAAGATACCAGACATTT	0/8	n	n
	CCGAAACCACAGATAATATATGGGGCACGAGGGACAAGATACCAGACATTT	0/8	n	n
**Clone 8**	CCGAAACCACAGATAATATATGGGGCACGAGGGACAAGATACCAGACATTT	0/8	n	n
	CCGAAACCACAGATAATATATGGGGCCGGCGGTACAGGATACCAGACATTT	4/8	n	y
**Clone 12**	CCGAAACCACAGATAATATATGGGGCACGAGGGACAAGATACCAGACATTT	0/8	n	n
	CCGAAACCACAGATAATATATGGGGCCGGCGGTACAGGATACCAGACATTT	4/8	n	y
**Clone 17**	CCGAAACCACAGATAATATATGGGGCACGAGGGACAAGATACCAGACATTT	0/8	n	n
	CCGAAACCACAGATAATATATGGGGCCGGCGGTACAGGATACCAGACATTT	4/8	n	y
**Clone 30**	CCGAAACCACAGATAATATATGGGGCACGAGGGACAAGATACCAGACATTT	0/8	n	n
	CCGAAACCACAGATAATATATGGGGCCGGCGGTACAGGATACCAGACATTT	4/8	n	y
Clone 31	CCGAAACCACAGATAATATATGGG-CACGAGGGACAAGATACCAGACATTT	0/8	y	n
	CCGAAACCACAGATAATATATGGGGCACGAGGGACAAGATACCAGACATTT	0/8	n	n
Clone 32	CCGAAACCACAGATAATATATGGGGCACGAGGGACAAGATACCAGACATTT	0/8	n	n
	CCGAAACCACAGATAATATATGGGGCACGAGGGACAAGATACCAGACATTT	0/8	n	n
Clone 33	CCGAAACCACAGATAATATATGGG-CACGAGGGACAAGATACCAGACATTT	0/8	y	n
	CCGAAACCACAGATAATATATGGGGCACGAGGGACAAGATACCAGACATTT	0/8	n	n
Clone 34	CCGAAACCACAGATAATATATGGGGCACGAGGGACAAGATACCAGACATTT	0/8	n	n
	CCGAAACCACAGATAATATATGGGGCACGAGGGACAAGATACCAGACATTT	0/8	n	n
Clone 35	CCGAAACCACAGATAATATATGGGGCACGAGGGACAAGATACCAGACATTT	0/8	n	n
	CCGAAACCACAGATAATATATGGGGCACGAGGGACAAGATACCAGACATTT	0/8	n	n
Clone 36	CCGAAACCACAGATAATATATGGGGCACGAGGGACAAGATACCAGACATTT	0/8	n	n
	CCGAAACCACAGATAATATATGGGGCACGAGGGACAAGATACCAGACATTT	0/8	n	n
**Clone 38**	CCGAAACCACAGATAATATATGGGGCACGAGGGACAAGATACCAGACATTT	0/8	n	n
	CCGAAACCACAGATAATATATGGGGCCGGCGGTACAGGATACCAGACATTT	4/8	n	y
	CCGAAACCATA---------------------------------------TGAATTTCAATTATT	0/8	y	y
Clone 43	CCGAAACCACAGATAATATATGGGGCACGAGGGACAAGATACCAGACATTT	0/8	n	n
	CCGAAACCACAGATAATATATGGGGCACGAGGGACAAGATACCAGACATTT	0/8	n	n

Figure S3. MiSeq sequencing results for all clones analyzed. This is an extended version of Figure 1, showing sequencing results for the 8 randomly chosen clones negative for HDR editing in the specific PCR test, in addition to the 6 clones found to be positive.

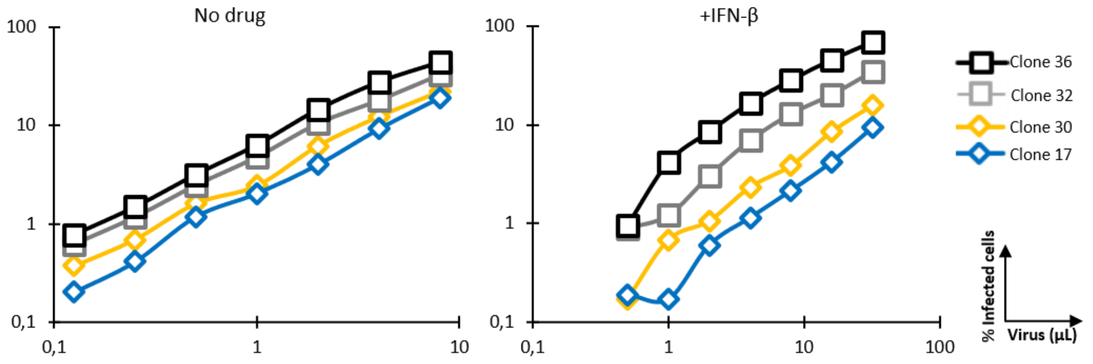

Figure S4. Permissiveness of HDR-edited clone 17 to HIV-1NL-GFP infection. Jurkat clonal cell populations were analyzed for permissiveness to HIV-1 infection. Clones 17 and 30 are monoallelically edited to express R332G-R335G TRIM5α whereas clones 32 and 36 are unedited. Cells were infected with increasing doses of HIV-1NL-GFP in the presence or not of IFN-β. The percentage of cells expressing GFP was determined by FACS two days later.
